# Empowering Self-Management of Chronic Low Back Pain Among Spanish and Cantonese Speakers in the United States

**DOI:** 10.7759/cureus.97090

**Published:** 2025-11-17

**Authors:** Patricia Zheng, Emilia De Marchis, Jan Yeager, Karina Del Rosario, Masato Nagao, Tigist Belaye, Angela Gallegos-Castillo, Lei-Chun Fung, Adrian Vallejo, Amy Kuang, David Gendelberg, Ashraf El-Naga, Jeffrey Lotz, Conor O'Neill

**Affiliations:** 1 Physical Medicine and Rehabilitation, University of California San Francisco, San Francisco, USA; 2 Family Medicine, University of California San Francisco, San Francisco, USA; 3 Clinical Innovation Center, University of California San Francisco, San Francisco, USA; 4 Orthopedic Surgery, University of California San Francisco, San Francisco, USA

**Keywords:** chronic non-specific low-back pain, focus group discussions, human-centered design, language barrier, self-management

## Abstract

Background: Chronic low back pain (cLBP) is a major cause of disability worldwide and disproportionately affects patients with limited English proficiency (LEP) who face linguistic, cultural, and socioeconomic barriers to care. Spanish- and Cantonese-preferring populations in the U.S. often struggle with limited access to culturally appropriate resources, highlighting the need for patient-centered approaches.

Methods: We conducted a qualitative study at an urban, academic-affiliated county hospital between January and June 2024. Focus groups were facilitated by bilingual, bicultural researchers. The study objective was to explore the priorities, barriers, and self-management preferences of Spanish- and Cantonese-preferring patients with the human-centered design (HCD) approach. Transcripts were translated, reviewed, and analyzed inductively to identify key themes. Institutional Review Board (IRB) approval to interview fifteen participants was obtained prior to study initiation.

Results: Fifteen Spanish- and Cantonese-preferring patients with cLBP participated in six focus groups. Participants reported 6.82 on the numerical rating scale of pain (NRS) (SD 2.49), with 71% of Spanish-speaking and 78% of Cantonese-speaking participants reporting 10/10 “complete trust” in their healthcare providers. Thematic analysis revealed four key themes: the need for empathic, tailored educational supports; desire for plans that reflect social and economic realities; recognition of mental health and social isolation as contributors to pain; and a need for clearer, trustworthy guidance on self-management. Participants preferred plain-language, video-based resources and support in understanding cLBP causes and management. Across both groups, patients expressed confusion about trustworthy information sources and called for clinician-vetted guidance and clearer explanations of self-management strategies.

Conclusions: Spanish- and Cantonese-preferring patients with cLBP face significant barriers to self-management and would benefit from culturally and linguistically appropriate resources. This study highlights the need for healthcare systems to develop and deliver tailored, accessible self-management support materials that address the unique challenges faced by patients with LEP.

## Introduction

Chronic low back pain (cLBP) is one of the leading causes of disability worldwide, affecting a diverse range of populations across different socioeconomic and cultural contexts [1-3]. The burden of cLBP is particularly high among marginalized groups, including those who face language, racial, and cultural barriers to healthcare [4-19]. Language preference plays a critical role in shaping access to care, treatment outcomes, and patient satisfaction [20-22]. For patients with limited English proficiency (LEP) in the United States, these barriers are further compounded by socioeconomic factors such as poverty, limited education, and restricted access to resources [23,24]. These structural inequities contribute to the disproportionately high levels of cLBP-related disability and dissatisfaction with care in these communities [19,25].

Current approaches to cLBP management emphasize patient engagement through self-management strategies, which include physical therapy, exercise, lifestyle changes, and psychological support [26-28]. However, existing self-management programs often fail to account for the unique challenges faced by patients with LEP [29,30]. These patients may struggle to understand care instructions, lack access to educational materials that are culturally and linguistically appropriate, and face socioeconomic challenges that hinder their ability to prioritize self-care [24,30,31]. Moreover, much of the existing literature on cLBP care overlooks the specific needs of populations with LEP, particularly in the context of low-income, urban environments [30]. This gap in research underscores the need for a more nuanced understanding of how to support these patients in managing their cLBP.

Human-centered design (HCD) is a promising approach to reducing disparities in cLBP outcomes by centering on patients’ lived experiences and creating self-management interventions tailored to their specific needs [32]. Unlike traditional top-down healthcare models, HCD engages patients as co-creators in the design process, ensuring that solutions are not only culturally and linguistically appropriate but also practical and feasible within the constraints of their daily lives. HCD has been successfully used to improve health literacy and confidence in health-seeking behaviors among historically and currently excluded populations [33-35].

This qualitative study aimed to explore the cLBP self-management priorities of Spanish- and Cantonese-speaking patients at an urban, academic-affiliated county hospital. Using an HCD approach, we identified how existing self-management resources could be optimized to better meet the needs of these populations.

## Materials and methods

Study design and setting

The study was conducted at the Zuckerberg San Francisco General Hospital, San Francisco, CA, USA. This qualitative study used an HCD approach to explore the self-management priorities of Spanish- and Cantonese-preferring patients with cLBP using thematic analysis of focus groups conducted in participants’ preferred language. Key themes in participants’ experiences with cLBP care, barriers to self-management, and preferences for educational materials were identified as informed by the Braun and Clarke framework [36]. We adhered to the Consolidated Criteria for Reporting Qualitative Research (COREQ) guidelines throughout the study design, data collection, and analysis processes [37].

This study was approved by the Institutional Review Board (#23-38969, approved July 21, 2023) and took place at an urban, academic-affiliated county hospital. All participants provided written informed consent prior to their involvement in the study.

Participant recruitment

We recruited a convenience sample of adult patients who had clinic visits between January and May 2024. Patients were eligible for participation if they met the NIH Research Task Force criteria [38] for cLBP. As previously described, this was defined as a current self-report of cLBP (pain between the lower posterior margin of the rib cage and the horizontal gluteal fold), which has persisted for at least the past three months and has resulted in pain on >50% of days in the past six months. Participants were included if they reported preferring to speak either Spanish or Cantonese during healthcare interactions. We identified patients via chart review and clinician referrals, and those who met the inclusion criteria were contacted by phone or in person in their preferred language by bilingual study team members or using a certified phone interpreter to ensure accuracy of interpretation. Patients were excluded if they had cognitive limitations that made it difficult for them to engage in the focus group. We welcomed participants who had a variety of spine treatments, including prior surgery.

Eligible patients were invited to participate in focus groups and informed that participation was voluntary, would not impact their care, and would include a financial incentive of $125 for their time. Participants were invited to attend one, two, or all three focus groups in their preferred language, based on their availability.

Data collection

We conducted a total of six focus groups (three each for Spanish- and Cantonese-preferring participants) from February to June of 2024. Focus groups were held within a conference room in an urban, academic-affiliated county hospital on the same campus as where they were receiving back pain care. Only participants and researchers were present. Each session lasted approximately 90 minutes and was facilitated by native bilingual, bicultural research team members (AGC: native Spanish speaker, and LCF: native Cantonese speaker) with more than 20 years of experience in community outreach and prior experience in medical research. Semi-structured guides were developed as advised by the research team members (PZ, EHD, JY, TB). These guides were initially developed in English and were adapted as culturally and linguistically appropriate from feedback from the bilingual, bicultural focus group facilitators. The guides were translated into Spanish and Cantonese and back-translated by the bilingual, bicultural members of the team to check for accuracy (Appendix 1 for focus group guides). Focus group facilitators had no prior relationship with participants and explained their reasons for participating in the research at the start of the sessions.

**Figure 1:**
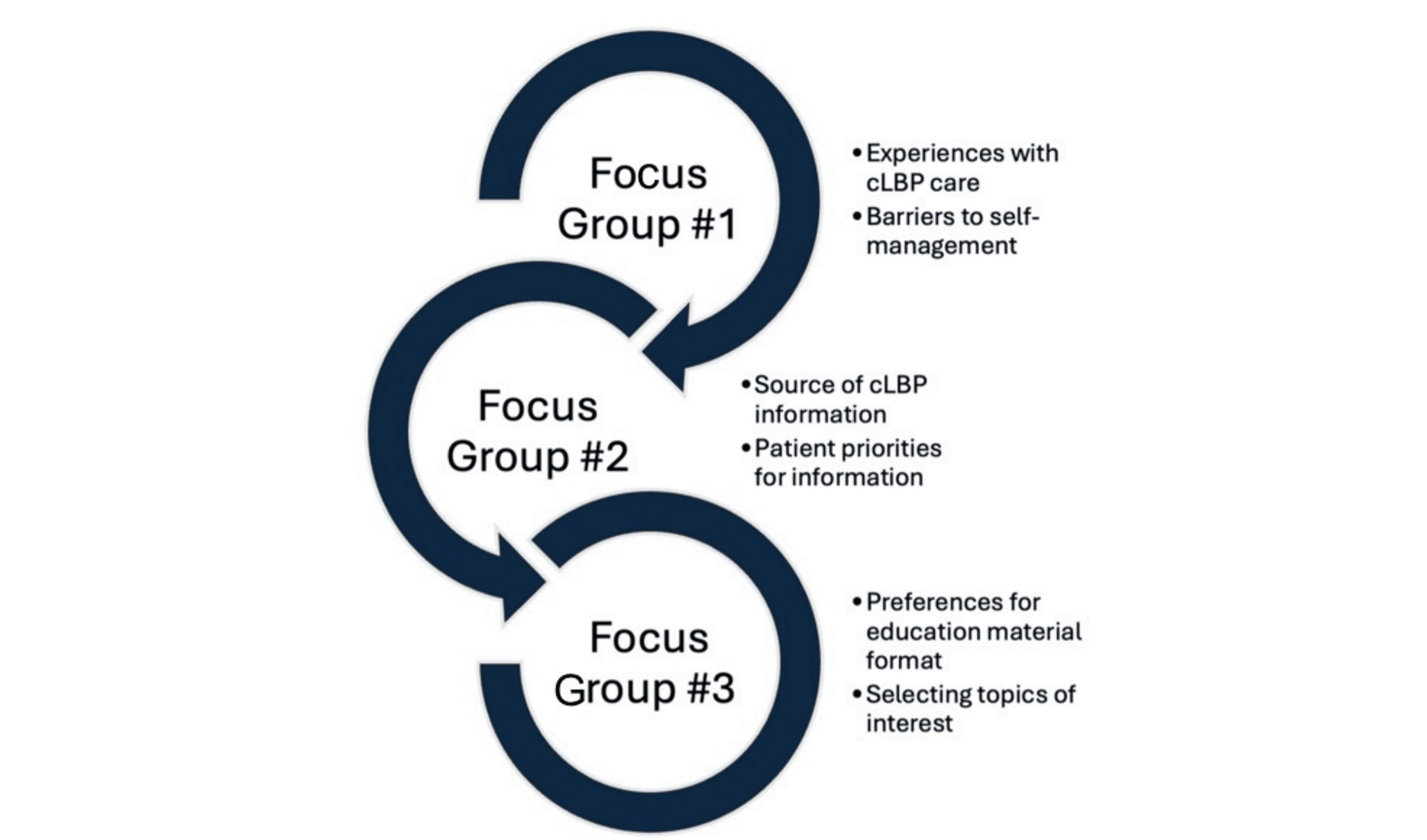
Overview of the topics of the three sequential focus groups cLBP: chronic low back pain

Focus groups followed a sequential structure. Earlier sessions (focus groups 1 and 2) aimed to identify patient priorities and barriers to self-management, including probing on participants’ prior experiences with cLBP care and barriers to self-management. The final session (focus group 3) centered on identifying patient preferences regarding the format and content of self-management support materials, including gathering their suggestions for improving educational resources (Figure 1).

All focus groups were audio recorded, transcribed verbatim, and translated into English by the company TranscriptionWing (Civicom®, Inc., Greenwich, CT, USA). Translations underwent a back-translation process by bilingual and bicultural study team members (AK: native Cantonese speaker, AGC, LCF), including the bilingual and bicultural focus group facilitators, to ensure accuracy and cultural relevance. Transcripts were reviewed by the focus group leads for accuracy and conceptual equivalence. Transcripts were not returned to participants for comments. Field notes were made after focus groups.

Participants self-completed a brief survey (Appendix 2 for survey items and citations). This included demographic information, household income, education level, language preferences, the Pain, Enjoyment of Life, and General Activity (PEG) scale [39], the numerical pain rating scale [40], and a 10-point Likert scale measure of trust in their healthcare providers. Surveys were available in participants' preferred languages. Questions were harmonized with those used in other trials [41].

Data analysis

We conducted thematic analysis as informed by Braun and Clarke’s approach [36]. Codes were developed to focus on identifying participant priorities for self-management support, barriers to care, and design preferences for educational materials. Coding was inductive and iterative, performed by two trained qualitative researchers (EHD and JY), with reflexive memoing and regular analytic discussions to interrogate assumptions and enhance interpretive rigor. Consistent with reflexive thematic analysis, we did not use ‘saturation’ as a stopping rule; instead, we judged analytic sufficiency by the coherence and stability of key themes. Microsoft Excel (Microsoft Corporation, Redmond, Washington, United States) was used to tabulate results.

In our analyses, we highlight differences, when identified, between the Spanish- and Cantonese-preferring focus groups, such as differing views on pain management efficacy and experiences of resource availability. Participants did not provide feedback on the findings.

## Results

Participant demographics

**Figure 2:**
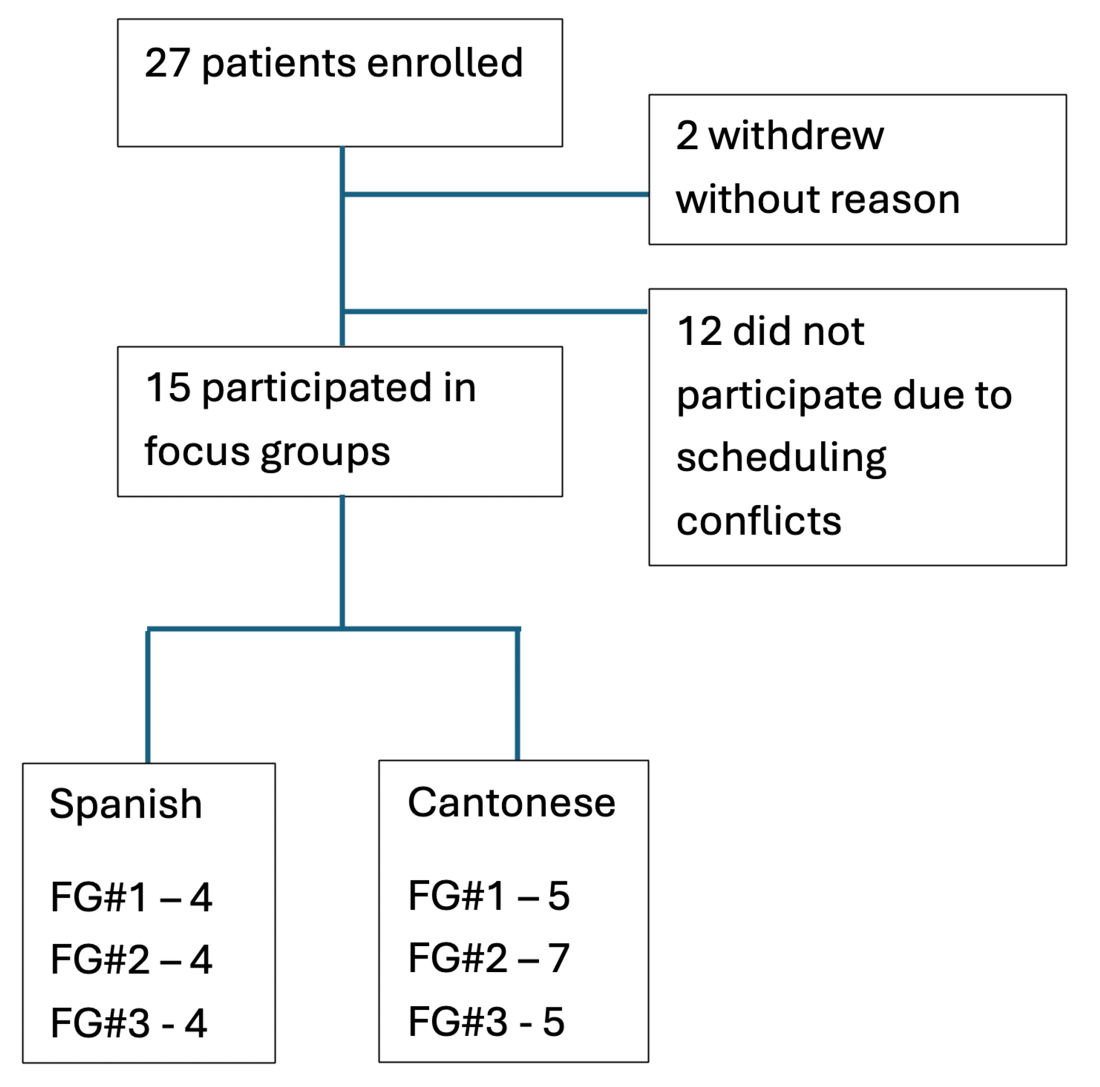
Flow diagram of PROTECT patients Flow diagram demonstrating recruitment of emPowering chRonic lOw pain patients To rEduCe dispariTies (PROTECT) patients and participation in focus groups (FG).

A total of 27 unique patients enrolled in the study; 15 participated in focus groups. Two patients withdrew after initial consent and did not provide a reason. Twelve patients enrolled but did not participate due to scheduling conflicts (Figure 2). Of the focus group participants, eight (53%) identified as Spanish-speaking, and seven (47%) identified as Cantonese-speaking. The mean age of participants in the Spanish-speaking group was 55.1 (SD 12.2) years, while the mean age in the Cantonese-speaking group was 69.0 (SD 2.0) years. Participants reported moderate to severe pain levels, with an average score of 6.82 on the NRS (SD 2.49). Participants also expressed high levels of trust in their healthcare providers, with 71% of Spanish-speaking and 78% of Cantonese-speaking participants reporting 10/10 “complete trust” in their healthcare providers (Table 1).

**Table 1:** Demographics of focus group participants 1: Answered "How hard is it for you (and your family) to pay for the very basics like food, medical care, and heating?” Answering “very hard,” “hard,” or “somewhat hard” was considered positive for financial difficulties, versus “not hard at all.” 2: Answered “How much do you trust your health care provider(s) at this clinic?” Answering 10/10, “Completely,” on a 10-point Likert scale was considered “complete trust.” * All others answered “don’t know” NRS: numerical rating scale of pain; FG: focus group

Demographics	Spanish-speaking				Cantonese-speaking			
	Total	FG#1	FG#2	FG#3	Total	FG#1	FG#2	FG#3
# of participants	8	4	4	4	7	5	7	5
Age (mean; SD)	55.1; 12.24	49.0; 14.02	55.3; 7.27	58.0; 11.11	67.3; 4.82	69.0; 2.0	67.3; 4.82	66.6; 5.55
#/% Female	5 (62.5%)	2 (50.0%)	2 (50.0%)	4 (100.0%)	4 (57.1%)	3 (60.0%)	4 (57.1%)	3 (60.0%)
$50,000 household income or less (#/%)	6 (75.0%)*	3 (75.0%)*	2 (50.0%)*	2 (50.0%)*	7 (100.0%)	5 (100.0%)	7 (100.0%)	5 (100.0%)
High school graduate or less (#/%)	5 (62.5%)	2 (50.0%)	2 (50.0%)	4 (100.0%)	7 (100.0%)	5 (100.0%)	7 (100.0%)	5 (100.0%)
Financial difficulties (#/%)^1^	3 (37.5%)	2 (50.0%)	2 (50.0%)	0 (0.0%)	6 (85.7%)	4 (80.0%)	6 (85.7%)	5 (100.0%)
NRS (mean; SD)	6.9 (2.4)	6.3 (2.9)	7.8 (2.6)	7.8 (1.7)	6.4 (1.8)	6.2 (2.2)	6.4 (1.8)	6.0 (1.0)
“Complete” trust in healthcare providers (#/%)^2^	6 (75.0%)	3 (75.0%)	2 (50.0%)	3 (75.0%)	6 (85.7%)	5 (100.0%)	6 (85.7%)	4 (80.0%)

Themes and key findings

Four main themes emerged from the focus groups related to patient priorities for self-management support and preferences for educational materials. These themes were consistent across both Spanish- and Cantonese-speaking participants, although specific concerns varied slightly between the two groups (Table 2).

**Table 2:** Major themes identified through focus groups cLBP: chronic low back pain

Theme	Quotes
Theme 1: Need for empathic, tailored educational supports	“In physical therapy, a therapist sees me and just prescribed some movements without any explanation." “They are very kind, they look for Chinese information to show me because I told them I don’t know how to read the full stack of documents, they tried to look for a Chinese translator for me, they say they really want to help me to look for a translator, but they are unable to do so. Very helpless.”
Theme 2: Need for self-care plans that fit into daily life	"The gym is very expensive, and I don't have time." "I think the video would be better because if I'm at work and have a five-minute break and I'm feeling bad, if I have my phone, I could play it and start doing my exercises." "I don’t usually walk outside because it’s not safe around my house area. Now they assault you, so I do not dare to leave my house."
Theme 3: Recognizing the mental and physical health impact of cLBP	"I think the most helpful method is to increase the exercise that we can do by ourselves, exercise that can we work on ourselves. Including taking care of the mood too." "I think we lack both things. The ability to exercise, of any kind, and the mental health aspect, having a therapist in charge, because this affects our emotions, it affects us a lot."
Theme 4: Need for clearer guidance on self-management strategies	"The doctor can introduce which video to watch and give suggestions. These suggestions will be professional because not every exercise is suitable for everyone." “The thing is, if I don't feel that you truly have a command of what you're telling me, I don't trust you. Then someone else comes and tells me something different, which seems better than what they told me, and everyone says something different." "I see 500 different exercises on YouTube (Google LLC, Mountain View, California, United States), and I don't trust any of them."

Theme 1: Need for Empathic, Tailored Educational Supports

Participants across both Spanish- and Cantonese-language focus groups expressed dissatisfaction with current cLBP care plans, often describing them as unclear, ineffective, or not aligned with their personal experiences. Participants reported a lack of understanding about the causes of their pain and how treatments such as physical therapy or exercise could help.

Cantonese-speaking participants emphasized the “incurable” nature of their pain and voiced concern that self-management strategies like exercise or physical therapy would be ineffective. Some participants conflated prescription medication with self-care and did not view non-pharmacologic interventions as helpful. One participant specifically voiced, "Don't have any (self-care); I believe in my medication." As one Cantonese-speaking participant noted, "It (physical therapy) was not helpful. Cannot cure my nerve pain. Regardless of what is done, my nerve was damaged. It is incurable."

Spanish-speaking participants highlighted the need for better-quality educational materials, with many expressing frustration that information provided by clinicians was either confusing or not helpful. There was a strong desire for clear explanations of their diagnoses and why particular treatments were recommended. As a Spanish-speaking participant noted, "My primary care doctor referred me to physical therapy. In physical therapy, a therapist saw me and just prescribed some movements without any explanation."

Participants in both focus groups strongly preferred self-management materials that were visually engaging, easy to understand, and available in their preferred language. Short videos were the most requested format. A Spanish-speaking participant voiced, "I never watched many videos because of the time issue, because to get people to pay attention, it should be short periods of time." Participants emphasized the need for clear, simple instructions with accompanying visual aids, such as diagrams or pictures. One Cantonese-speaking participant shared, "Have simple pictures. Easier choice of words with a picture." Both groups highlighted the need for material that is culturally relevant and uses empathetic communication and a recognizable “face” or trusted authority to deliver the information.

Theme 2: Need for Self-Care Plans That Fit Into Daily Life

Participants, particularly those in the Spanish-speaking groups, emphasized the difficulty of incorporating self-care practices into their daily routines, especially given their social and economic circumstances. Many participants reported working multiple jobs, lacking access to exercise facilities, or facing financial constraints that limited their ability to engage in recommended activities like yoga or physical therapy. One Spanish-speaking participant noted, "The gym is very expensive, and I don’t have time."

Cantonese-speaking participants also reported challenges in accessing safe environments for exercise due to fear of being attacked in their communities, reflecting broader concerns about personal safety. One Cantonese-speaking participant noted, "I don’t usually walk outside because it’s not safe around my house area. Now they assault you, so I do not dare to leave my house."

Both groups expressed a preference for self-management resources that were easy to follow and accessible at any time, such as short videos or simple written guides. Spanish-speaking participants favored videos that could be accessed via smartphone during brief breaks, while Cantonese-speaking participants requested written materials with clear visuals to help with understanding.

Theme 3: Recognizing the Mental and Physical Health Impact of cLBP

Both Spanish- and Cantonese-speaking participants discussed the profound impact of cLBP on their mental health. One Spanish-speaking participant noted the importance of "the mental health aspect, having a therapist in charge, because this affects our emotions; it affects us a lot." Feelings of isolation were common, with participants expressing a desire for their care teams to facilitate connections with others experiencing similar challenges. Group visits, support groups, and community-based exercise programs were suggested as potential solutions to reduce isolation and provide mutual support. A Spanish-speaking participant noted, "Yes, it would be good to have courses or workshops that you can attend. Maybe one or two times a week; that would be something I would like." Cantonese-speaking participants also recognized the need for more continuity in their care, noting the turnover among clinicians. One participant reported feeling isolated after "they suddenly changed the doctor (on me), and he is not caring, as the previous doctor was." I just want my original doctor, but I don’t have any information on how to find him." 

Theme 4: Need for Clearer Guidance on Self-Management Strategies

Participants across both groups expressed difficulty in determining which materials or online resources were trustworthy. Many noted the overwhelming amount of conflicting information available online and stressed the importance of receiving guidance directly from clinicians. As a Cantonese-speaking participant noted, "The doctor can introduce which video to watch and give suggestions. These suggestions will be professional because not every exercise is suitable for everyone." Spanish-speaking participants also noted feeling unsure about which exercises were appropriate for their specific condition: "I see 500 different exercises (on YouTube, Google LLC, Mountain View, California, United States), and I don't trust any of them." Furthermore, a Spanish-speaking participant voiced, "There is a lot of information on YouTube or Facebook (Meta Platforms, Inc., Menlo Park, California, United States), and when you start searching, there's more and more. But, when I talk about quality information, it's because there are all kinds of people and all kinds of opinions there, but it would be good if there was a specialist who could tell you if, out of all that information, this is how it is or that's how it is."

Despite reporting high levels of trust in their healthcare providers on surveys, both groups commented on skepticism about self-management strategies and confusion about how to approach their care, given differences in opinions from different sources. One Spanish-speaking participant voiced, “The thing is, if I don't feel that you truly have a command of what you're telling me, I don't trust you. Then someone else comes and tells me something different, which seems better than what they told me, and everyone says something different.” Both groups called for clearer explanations of the causes of their pain, what self-management entails, and what they could realistically expect from different treatment options. One Spanish-speaking patient voiced, "All these things exist and are in videos and such, but (the physicians) should tell me what's good for me. I don't know if doing this or that will help me; I don't know that."

## Discussion

This study aimed to explore the self-management needs and priorities of Spanish- and Cantonese-speaking patients with cLBP at an urban county hospital. Using an HCD approach, we identified key barriers to care and opportunities to improve self-management resources for our study populations. The findings reveal that patients faced significant challenges in accessing appropriate resources, understanding care plans, and integrating self-management practices into their daily lives. Our results suggest the need for more culturally tailored, accessible, and comprehensive self-management support systems and build upon prior research on the need for accessible and patient-centered cLBP self-management support [42,43].

One of the major findings of this study was the pervasive sense of skepticism and confusion regarding cLBP diagnoses, particularly among Cantonese-speaking participants. Despite reporting high levels of trust in their healthcare providers, participants expressed frustration with care teams who failed to explain the underlying causes of their pain or how prescribed treatments would help alleviate it. This is a common problem with cLBP care, as 80 to 90% of cLBP is considered nonspecific, without specific identifiable causes [44].

While in other populations, patients report existing acceptance that self-care is an integral part of cLBP management [45], in our participants, the lack of clarity contributed to a perception that self-care, particularly physical therapy, was ineffective, leaving patients feeling that their pain was “incurable," as stated by one Cantonese-speaking participant. Studies are needed to elucidate whether patients with LEP have differing perceptions of self-management, and effort should be dedicated to developing tailored information for these populations.

Education on cLBP causes and self-management strategy is widely supported by multiple professional society practice guidelines [46,47]. A review of 41 studies on the health information needs of low back pain patients showed that patients want information about the causes of their condition, its prognosis, and self-management strategies [48]. Our focus group participants echoed these sentiments. Specifically, our participants called for clear guidance on what self-management entails, including the role of physical activity, the expected outcomes of different treatments, and how to safely perform exercises. There was a consistent demand for clear, consistent information presented in understandable language. Multiple focus group participants referenced looking online for available health information. Prior research found that most cLBP-related information online is classified as advertising with variable quality of information [49]. As a result, both groups stressed the need for materials that have been “verified” by trusted health professionals. Patients with LEP may also be receptive to emerging digital health interventions that have been found to have some success in supporting decision-making and delivering care for cLBP [50].

The language barrier experienced by cLBP patients with LEP creates unique needs and may contribute to difficulties accessing self-management care. Studies have shown that, in general, non-English speakers have worse outcomes than those who are English-proficient [51]. Studies have highlighted specific strategies to help patients overcome linguistic and cultural barriers to seeking care. For example, among Spanish-speaking populations in the United States, the use of community health workers, known as promotores, and multimedia tools like photo comics (fotonovelas) that are culturally and linguistically adapted has been highlighted as an effective approach [52]. Analysis of efforts to develop cancer screening educational materials for Cantonese speakers emphasized the importance of incorporating sociocultural values and health beliefs [53]. Similarly, our participants stressed the importance of having material presented in their native languages in clear and understandable terms. Our study revealed that these patients specifically asked for clinician-vetted materials in a variety of formats, such as short videos with graphics.

Our findings also highlight potential ways social determinants of health (SDOH) may make it difficult for participants with LEP to pursue self-management. Spanish-preferring participants specifically reported dissatisfaction with self-care recommendations, noting that the recommendations were difficult to implement given their socioeconomic constraints. Participants, many of whom were working multiple jobs, also highlighted the challenge of finding the time and resources to follow prescribed self-management activities. Additionally, they raised concerns about how mental health and competing life demands, such as financial and familial responsibilities, further hindered their ability to prioritize self-care. Educational and self-care information should account for the target audience’s specific socioeconomic constraints and consider how to make care low-cost and accessible in a limited timeframe.

While walking programs have been found to be a low-cost, effective, and “easily accessible” form of intervention for cLBP [54], Cantonese-preferring participants commented on concerns over safety to be able to walk outside for exercise, given the recent rise of hate crimes and negative biases against Asian American individuals after the start of the COVID-19 pandemic [55]. Socioeconomic status has been strongly associated with cLBP outcome [56], and our studies highlight how the SDOH of our participants may impact their ability to adhere to self-management. For participants who have concerns about safety outside of their home, it's important to consider home-based alternatives.

Another major theme was the recognition of how cLBP affected participants' mental health and contributed to feelings of isolation. Feelings of isolation may be particularly notable in populations with language barriers, which may make it more difficult to find social connections [57]. Both groups expressed a desire for their care team to serve as a point of connection, helping them link with other patients experiencing similar challenges. Group-based care, including workshops, exercise classes, and support groups, was suggested as a means of reducing isolation and promoting mental and emotional well-being. The importance of addressing mental health as a component of self-management was particularly salient for Spanish-speaking participants, who called for greater access to mental health resources and social support services. Indeed, group-based cognitive behavioral treatment has been shown to improve back pain outcomes and be cost-effective in other countries [58]. Support groups without clinician involvement have also been found to be helpful in increasing functional ability and activity and decreasing the need for healthcare seeking [59]. These types of interventions are not widely available in our practice setting. Healthcare systems should explore ways to collaborate with community organizations and patients themselves as part of the HCD process to create safe, culturally relevant spaces for self-management, such as group-based therapy or peer support programs.

This study is among the first to explore the specific self-management needs of Spanish- and Cantonese-speaking patients with cLBP, populations that have historically been underserved in the healthcare system. Our findings underscore the need for tailored self-management resources that not only address linguistic barriers but also consider cultural, social, and economic factors. Future interventions aimed at reducing cLBP disparities should prioritize the development of culturally appropriate educational materials, including short videos, podcasts, and written guides that are accessible, easy to understand, and actionable.

Limitations

This study has a number of limitations. First, this study was done at a single urban academic hospital with a convenience sample of patients who prefer to speak Spanish or Cantonese. The views expressed may not reflect those of patients with LEP in other settings. Second, the study is subject to selection and social desirability bias. From surveys, we know that there were demographic differences between participants in the Spanish versus the Cantonese language focus groups. Spanish-preferring participants were overall younger and active in the workforce; Cantonese-preferring participants tended to be older and out of the workforce. These differences may reflect some of the differences in perspectives between the two sets of focus groups that could have been more related to age and life experience, not true differences related to language or culture. Third, patients from our study setting who declined to participate may have perspectives not captured by the study population. However, across both focus groups, participants expressed similar perspectives, decreasing the likelihood of selection bias. Fourth, we acknowledge not sharing transcripts back with patients, not assessing for saturation formally, and not member checking present limitations. As a qualitative study, the goal was to capture insights from a specific patient population in our study setting to inform the development of tailored self-management educational material [60]. Further refinement of self-management materials should strive to capture input for a broader patient population.

## Conclusions

In conclusion, this study highlights the significant barriers that Spanish- and Cantonese-speaking patients face when attempting to self-manage their cLBP. The findings point to a pressing need for culturally sensitive, accessible, and practical self-management support systems that address both the physical and mental health challenges of living with cLBP. Future work should focus on developing and evaluating interventions that can reduce cLBP disparities by enhancing patient education, fostering social connections, and providing clear, reliable, and motivating self-management resources through HCD.

## Appendices

Appendix 1

Focus Group Guides

Draft Patient Focus Group Interview Guide Phase 1A (Discover)

Study Title: emPowering chRonic lOw pain patients To rEduCe dispariTies (PROTECT)

Intro

I’m going to start by asking you some questions about your prior experiences with your low back pain, focusing a lot on the things you do or have done in the past to manage your pain on your own, and what happened as a result.

1. How long have you had low back pain? Tell me about the care you have received for your back pain over time. 

2. What do you know about managing your pain on your own, that is, when not in the clinic?

            Probes:

- What types of self-management do you know about that can improve your pain?

            - Where do you go to access self-management material/resources for improving low back pain?

            - Are you able to find information in your preferred language?

- Why do you think self-management is recommended for your low back pain?

- What ways have you been taught to self-manage your low back pain? Who taught you this?

- Was this taught to you in a way that was easy to understand? How or how not?

- Were you given resources you could use after leaving the clinic? E.g., were you ever given resources you needed, a computer/tablet, or internet to use? And did you have the resources to use them?

3. How do you currently treat/manage your pain when you experience it? Walk me through what you do on your own, or with the assistance of friends/family, when you’re experiencing back pain?

Probes:

- What makes it hard to do these things?

- What makes it easy to do these things?

- How easy or difficult is it for you to manage your pain on your own?

- How well equipped do you feel in managing your pain on your own?

- How empowered do you feel to manage your pain on your own?

- How confident are you that you can manage your pain on your own?

- What techniques do you use to remember to do self-care/self-management of your pain?

- What is your current routine around self-management/care of your back pain?

- What types of self-management/care are a habit/routine for you?

- What things help you or might help you remember to do self-management/self-care?

4. What do you think will happen if you increase your self-care for your back pain on your own?

Probes:

- What would incentivize (help) you to do more self-care on your own?

- What are things that make you want, or not want, to manage your pain on your own?

- How do self-care activities conflict with other things going on in your life?

- Will you remember to do self-care/self-management activities?

- What things interfere with remembering to do self-management/care activities?

- What do you think is needed to help you keep doing self-care/self-management over time?

- What resources do you think are needed to do self-care/management of your back pain on your own?

What of these resources do you already have? Which ones do you think you need?

- What needs to happen for you to get these resources?

5. Among your peers/friends/family members who also have low back pain, what kind of things do they do to manage their pain?

Probes:

- How do you talk about pain management or exercises with your peers/friends/family?

6. As a patient, what do you see your role as in managing your symptoms?

7. What kind of feedback have you gotten before about self-management/care? How was it helpful/not helpful?

Probes:

- What types of positive reinforcement would you want for reaching your goals in self-management/care?

- What kinds of negative reinforcement have you had?

8. When you think about self-managing/caring for your back pain on your own, how does it make you feel? Does it bring up any emotions (good or bad)? Why do you think this is?

9. As a patient at this clinic, tell me more about your relationship with your health care team. Do you trust your health care team?

            Probes:

            - What makes you trust/not trust your health care team?

- Have you ever felt discriminated against by a member or members of your health care team? If you feel comfortable, can you tell me more about what happened and how it affected the medical care you were receiving?

10. What has been your experience accessing care?

            Probes:

            - What is your experience finding and accessing health care?

            - Do you feel you seek less care for cost reasons? Do you know how to find information about cost?

            - If you were referred to something like physical therapy or acupuncture, were you able to schedule and go to those visits? Why or why not?

Wrap up

Is there anything else you’d like to share with me?

What questions do you have for us?

Thank you for being willing to talk with me about these topics. It’s so helpful to hear how patients are managing their low back pain and what resources may be needed to help patients manage their pain.

Draft Patient Focus Group Interview Guide Phase 2B (Define)

Study Title: emPowering chRonic lOw pain patients To rEduCe dispariTies (PROTECT)

Intro

I’m going to start by asking you some questions about what kinds of materials you would be interested in to help you manage your low back pain on your own, and how you would like to get this information from your health care team.

1. What kinds of self-management or educational material have you received before about chronic low back pain and how to manage low back pain on your own?

            Probes:

- Was this information useful? Why? What made it useful?

 What not? What could have made it more useful?

            - Was this information provided in your preferred language?

- What format did you get this information in? For example, was it a handout? If yes, did someone go over the handout with you in the clinic? Or was it a video or information over the phone?

            - What about the format itself made the information helpful?

            - What about the format itself made the information less helpful?

2. What kinds of educational material have you gotten in the past from your health care team that was useful? What made it useful?

            Probes:

- If participants have never gotten useful information: Why do you think you’ve never gotten useful educational material? What would need to change to make educational material useful?

3. If you were to design educational material for yourself or other patients like yourself, what would it look like? E.g., would it be a handout with text, a handout with cartoons, a video, an email, a text message, a conversation with someone on the phone?

            Probes:

- Tell me more about why you would want the educational material to look that way-why do you think that would be helpful?

4. I’m now going to show you some examples of available educational material. Please let me know what you think is useful/not useful about these different materials.

Wrap up

Is there anything else you’d like to share with me?

What questions do you have for us?

Thank you for being willing to talk with me about this information. It’s so helpful to hear directly from patients about how we can make self-care materials more useful for patients, and what we need to do to make sure materials we develop will be appropriate for our patients who prefer to speak/read a non-English language.

Draft Patient Focus Group Interview Guide Phase 3C (Develop)

Study Title: emPowering chRonic lOw pain patients To rEduCe dispariTies (PROTECT)

Intro

I’m going to show you some material to help patients manage their back pain at home, and I’d like you to tell me what you think about each of them. Please say anything that comes to your mind-we really want any and all feedback, good or bad, so that we can make these materials more helpful for our patients.

Probes to include as appropriate as part of the Think Aloud method:

            (General probes)

·       Tell me more about why you think that.

·       What about this material is useful?

·       What about this material isn’t useful?

·       What makes it easy to use/review this material?

·       What makes it hard to use/review this material?

·       What parts are difficult to understand? What and why? How could it be improved?

·       What parts are easy to understand? What and why?

·       What do you like about this material?

·       What do you dislike about this material?

·       Can you describe what this material is saying in your own words?

·       I noticed that you hesitated a bit. Tell me what you were thinking.

·       How would you feel if your healthcare team gave you this material? Would you want someone to review it with you in the clinic, after the clinic, or just review it on your own? Why/why not?

Would it matter to you who reviewed it? Why?

·       If you were given this material, would you review it? Why/why not?  What would make you more likely to review it and use it?

·       How confident are you in your ability to use/review this material on your own?

·       What is missing?

·       What is distracting about this material?

For FG D only:

-       In our last discussion with patients we heard ___(insert themes from FGC)_____ and we made these changes: _______(insert changes made))_________.

-       Do you think that the changes made were helpful? Why/why not?

-       What additional changes do you think are needed to address prior patient concerns/feedback?

-       What additional changes do you suggest?

Wrap up

Is there anything else you’d like to share with me?

What questions do you have for us?

Thank you for being willing to talk with me about these materials. It’s so helpful to hear directly from patients about how we can make these materials more useful for patients.

Appendix 2

Survey Items and Citations

PROTECT Surveys and Rationale

All patients should complete the form after consent, before focus groups. Survey items were harmonized with those as per the Back Pain Consortium Research Program.1 All surveys are freely publicly available and do not require permission for use.

Sex

What was your sex at birth?

▢ Male

▢ Female

▢ Unknown

▢ Intersex

Gender Identity

What is your gender identity?

▢ Male

▢ Female

▢ Transgender man

▢ Transgender woman

▢ Genderqueer or gender nonconforming

▢ Unknown

▢ Other: _________  

Ethnicity

What is your ethnicity?

i.e., Are you of Hispanic or Latino origin?

▢ Hispanic 

▢ NOT Hispanic 

▢ Unknown

▢ Prefer not to answer

Race

What is your race?

“Race” is a person's self-identification with one or more cultural groups.

(Mark all that apply)

▢ Alaska Native

▢ Asian

▢ Black 

▢ Pacific Islander       

▢ White

▢ Unknown

▢ Prefer not to answer

Asian/PI Subgroup

Please indicate all that apply.

(Mark all that apply)

▢ Chinese

▢ Filipino

▢ Vietnamese

▢ Korean

▢ Japanese

▢ Asian Indian

▢ Samoan

▢ Chamorro

▢ Other Asian (e.g., Pakistani, Cambodian, Hmong, etc.)

Insurance payer

What type of medical insurance do you have?

(Mark primary one)

▢ Private insurance (HMO, PPO, etc.)

▢ Medicare

▢ Medicaid

▢ VA

▢ Indian Health Service

▢ National Health Care System (from outside of the US)

▢ No insurance/Self-pay

▢ Other

Financial Need

How hard is it for you (and your family) to pay for the very basics like food, medical care, and heating?

1 ▢ very hard

2 ▢ hard

3 ▢ somewhat hard

4 ▢ not very hard

5 ▢ don’t know

Employment

Status

What is your current employment status?

“Not employed” includes:

not employed outside of the house (i.e., keeping house), disabled, retired, and students

1 ▢ Full-time employment

3 ▢ Part-time employment

2 ▢ Not employed

Income

Which of the following categories best describes your total combined household income for the past 12 months?

a)    0 - $5,000

b)    $5,001 - $10,000

c)     $10,001 - $15,000

d)    $15,001 - $20,000

e)    $20,001 - $25,000

f)      $25,001 - $30,000

g)    $30,001 - $35,000

h)    $35,001 - $40,000

i)      $40,001 - $50,000

j)      $50,001 - $75,000

k)     $75,001 - $100,000

l)      $100,001 - $150,000

m)   $150,000 +

n)    Don't know

o)    Would rather not say

Trust in healthcare provider (self-designed)

How much do you trust your health care provider(s) at this clinic?  

1 Completely

2 

3 

4 

5 

6 

7 

8 

9 

10 

Not at all  

Pain, Enjoyment of Life and General Activity (PEG) Scale 2

What number best describes your pain on average in the past week?

No Pain 0 1 2 3 4 5 6 7 8 9 10 Pain as bad as  you can imagine

What number best describes how, during the past week, pain has interfered with your enjoyment of life?

Does not interfere 0 1 2 3 4 5 6 7 8 9 10 Unable to carry on  any activities

What number best describes how, during the past week, pain has interfered with your general activity?

Does not interfere 0 1 2 3 4 5 6 7 8 9 10 Completely interferes

To compute the PEG score, add the three responses to the questions above, then divide by three to get a final score out of 10.
